# Is CT Radiomics Superior to Morphological Evaluation for pN0 Characterization? A Pilot Study in Colon Cancer

**DOI:** 10.3390/cancers16030660

**Published:** 2024-02-04

**Authors:** Marta Zerunian, Ilaria Nacci, Damiano Caruso, Michela Polici, Benedetta Masci, Domenico De Santis, Paolo Mercantini, Giulia Arrivi, Federica Mazzuca, Pasquale Paolantonio, Emanuela Pilozzi, Andrea Vecchione, Mariarita Tarallo, Enrico Fiori, Elsa Iannicelli, Andrea Laghi

**Affiliations:** 1 Radiology Unit, Department of Medical Surgical Sciences and Translational Medicine, Sapienza University of Rome, Sant’Andrea University Hospital, Via di Grottarossa 1035–1039, 00189 Rome, Italy; marta.zerunian@uniroma1.it (M.Z.); ilaria.nacci@uniroma1.it (I.N.); michela.polici@uniroma1.it (M.P.); benedetta.masci@uniroma1.it (B.M.); domenico.desantis@uniroma1.it (D.D.S.); elsa.iannicelli@uniroma1.it (E.I.); andrea.laghi@uniroma1.it (A.L.); 2 Ph.D. School in Translational Medicine and Oncology, Department of Medical and Surgical Sciences and Translational Medicine, Faculty of Medicine and Psychology, Sapienza University of Rome, Via Giorgio Nicola Papanicolau–ang. Via di Grottarossa 1035, 00189 Rome, Italy; 3 Surgery Unit, Department of Medical Surgical Sciences and Translational Medicine, Sapienza University of Rome, Sant’Andrea University Hospital, Via di Grottarossa 1035–1039, 00189 Rome, Italy; paolo.mercantini@uniroma1.it; 4 Oncology Unit, Department of Clinical and Molecular Medicine, Sapienza University of Rome, Sant’Andrea University Hospital, Via di Grottarossa 1035–1039, 00189 Rome, Italy; giulia.arrivi@uniroma1.it (G.A.); federica.mazzuca@uniroma1.it (F.M.); 5 Department of Radiology, San Giovanni Addolorata Hospital Complex, Via dell’Amba Aradam 8, 00184 Rome, Italy; paolantoniopasquale@hotmail.com; 6 Pathology Unit, Department of Clinical and Molecular Medicine, Sapienza University of Rome, Sant’Andrea University Hospital, Via di Grottarossa 1035–1039, 00189 Rome, Italy; emanuela.pilozzi@uniroma1.it (E.P.); andrea.vecchione@uniroma1.it (A.V.); 7 Department of Surgery “Pietro Valdoni”, Sapienza University of Rome, Via Giovanni Maria Lancisi 2, 00161 Rome, Italy; mariarita.tarallo@uniroma1.it (M.T.); enrico.fiori@uniroma1.it (E.F.)

**Keywords:** colon cancer, radiomics, lymph node metastasis, lymph node morphology

## Abstract

**Simple Summary:**

Lymph node (LN) involvement is one of the most important prognostic factors for patients with colon cancer (CC). CT morphological analysis is not a reliable method to assess nodal status. Thus, the aim of this study was to assess the potential added value of radiomic features extracted from contrast-enhanced computed tomography (CECT) images compared to morphological features when assessing regional LNs in patients with pathologically confirmed stage I to stage IIC CC, in order to obtain a non-invasive preoperative tool.

**Abstract:**

The aim of this study was to compare CT radiomics and morphological features when assessing benign lymph nodes (LNs) in colon cancer (CC). This retrospective study included 100 CC patients (test cohort) who underwent a preoperative CT examination and were diagnosed as pN0 after surgery. Regional LNs were scored with a morphological Likert scale (NODE-SCORE) and divided into two groups: low likelihood (LLM: 0–2 points) and high likelihood (HLM: 3–7 points) of malignancy. The *T*-test and the Mann–Whitney test were used to compare 107 radiomic features extracted from the two groups. Radiomic features were also extracted from primary lesions (PLs), and the receiver operating characteristic (ROC) was used to test a LN/PL ratio when assessing the LN’s status identified with radiomics and with the NODE-SCORE. An amount of 337 LNs were divided into 167 with LLM and 170 with HLM. Radiomics showed 15/107 features, with a significant difference (*p* < 0.02) between the two groups. The comparison of selected features between 81 PLs and the corresponding LNs showed all significant differences (*p* < 0.0001). According to the LN/PL ratio, the selected features recognized a higher number of LNs than the NODE-SCORE (*p* < 0.001). On validation of the cohort of 20 patients (10 pN0, 10 pN2), significant ROC curves were obtained for LN/PL busyness (AUC = 0.91; 0.69–0.99; 95% C.I.; and *p* < 0.001) and for LN/PL dependence entropy (AUC = 0.76; 0.52–0.92; 95% C.I.; and *p* = 0.03). The radiomics ratio between CC and LNs is more accurate for noninvasively discriminating benign LNs compared to CT morphological features.

## 1. Introduction

Colorectal cancer (CC) is the third most frequently diagnosed cancer and the third deadliest malignancy for both genders combined [1]. The most important prognostic CC indicator is the pathological stage at presentation. A key role is played by the lymph node (LN) status, which determines the extent of surgery, the formulation of adjuvant therapy, and the postoperative survival rate of patients [2,3,4]. The Surveillance, Epidemiology, and END Results (SEER) Program observed overall survival rates at five years for colon cancer in stage I were 80–95%; stage II, 55–80%; stage III, 35–55% (involved regional LNs); and stage IV, <15% (distant metastases) [5].

Currently, surgery is the primary form of treatment for localized non-metastatic CC [6]. However, surgical treatment is invasive, expensive, and may expose CC patients to postoperative complications [7]. Furthermore, patients with suspected malignant LNs after local excision necessitate additional radical surgical treatment, including the excision of regional LNs [2]. In clinical practice, computed tomography (CT) is the preferred imaging study for CC staging before surgical resection and for follow-up [4]. However, CT imaging parameters for the assessment of lymph nodes’ status in CC remain a subject of debate, and the use of size and conventional morphological criteria revealed a limited diagnostic performance when identifying LN metastases, often overstaged [8,9,10]. Thus, a non-invasive additional support might improve the discrimination between benign and malignant LNs. In this contest, radiomics could be a useful imaging tool, there being the expectancy that it will influence treatment decision making and predict patient prognosis based on nodal microarchitecture, microenvironment, and heterogeneity by extracting quantitative features from volumetric LN segmentation [11,12,13,14].

In recent literature, only a few studies have investigated the role of radiomics in predicting LN involvement in patients with CC, with these studies having the highest goal of introducing predictive models of the overall risk of LN metastases that combine radiomics and preoperative clinical indicators. However, a comparison between the individual pathological status and the qualitative features of each single LN analyzed has not been explored so far [15,16]. In fact, to clearly identify which malignant lymph node found at pathology corresponds to a specific node detected at imaging is very challenging. To obtain that, a careful intraoperative identification of specific LNs previously mapped and suspected of being malignant at a preoperative CT would be required. This would be difficult to realize because it requires an in-depth dialogue between the radiologist, the surgeon, and the pathologist, which is very difficult across large prospective cohorts or in retrospective studies. With these assumptions, a feasible way to quantitatively study the inner LN’s structure could be to analyze extreme situations in which all LNs are pathologically positive or negative. Since the condition of all pathologically positive LNs is a rather rare condition, the analysis of N0 status is more accessible and can be the basis for testing the role of radiomics in the assessment of these structures. To the best of our knowledge, there are no studies which evaluate radiomics’ performance compared to morphologic criteria in LNs’ assessment using the CECT images of patients with histologically proven pN0 CC.

Thus, the aim of this study is to investigate the role of radiomics by comparing the radiomic and qualitative features when detecting histologically proven negative LNs on preoperative CECT scans of patients with CC (stage I to stage IIC). Subsequently, we aim to identify potential radiomic features that characterize lymph nodes’ benignity and compare them with the radiomic features of the primary tumor.

## 2. Materials and Methods

### 2.1. Patient Population and Study Design

This multicenter, retrospective observational study was in accordance with the Declaration of Helsinki and approved by the Ethics Committee of Sant’Andrea University Hospital (protocol code ref. nr. CE 6597/2021 on 12 January 2022). From a population of two-hundred and sixty-five patients admitted at BLINDED and BLINDED for CC from June 2020 to May 2023, eligible patients were selected according to the following inclusion criteria: (a) histological diagnosis of CC after surgery, (b) N0 status at pathology report according to AJCC 8th edition, and (c) availability of preoperative enhanced CT examination. Exclusion criteria were: (a) significant motion artifacts on CT scan, (b) examination of a specimen with fewer than 12 regional LNs, (c) unavailability of the portal-venous phase CT scan, (e) preoperative therapy (previous surgical intestinal resection, neoadjuvant treatment, or radiotherapy), and (f) synchronous other tumoral pathologies. For the validation cohort, another twenty patients were included and divided into pN0 (10 patients) and pN2 (10 patients) as per their LN status at pathological report. The inclusion/exclusion criteria were the same for the test cohort for pN0 patients, while in the pN2 sub-set patients were included with pathological report of pN2 status. Patients’ demographic characteristics, clinical findings, and laboratory results, including sex, age, comorbidities, and histopathological results, were retrieved from the internal hospital records.

### 2.2. CT Acquisition Technique

All patients underwent multiphases CT examination before surgery. CT scans were obtained by using 128-slices CT (GE Revolution EVO Slice CT Scanner, GE Healthcare, Milwaukee, WI, USA), with patients in supine position and the scans performed in cranio-caudal direction at end-inspiration. The acquisition protocol included unenhanced, arterial, and delayed phase acquired on the upper abdomen from diaphragm to the upper limit of iliac bones, and a porta-venous phase acquired from the clavicle bones to the pubic bones. In this study, portal-venous phase was selected for the radiomic analysis.

For each patient, the volume of contrast medium (CM) was tailored in accordance with lean body weight (LBW) [17,18]:

$$CM\;volume\;\left(mL\right)=\frac{0.7gI\;\times \;LBW\;\left(kg\right)}{CM\;concentration\;\left(mgI/mL\right)}$$

The administration of contrast medium bolus (Iomeprol 400 mg I/mL; Bracco, Milan, Italy) and the saline solution (40 mL) was performed with a flow rate of 3.0 mL/s by an antecubital venous access (18–20 gauge). The bolus-tracking method (Smart Prep, GE, Milwaukee, WI) was used for the contrast enhanced CT phases, setting within the abdominal aorta, at level of celiac tripod, a 150 HU-threshold region of interest. All patients were studied with unenhanced, late arterial (18 s from threshold achieved) and portal-venous phases (70 s from threshold achieved). CT scans were obtained by setting the following technical parameters: tube voltage 120 kV; tube current modulation 130–300 mAs by using SMART mA (GE Healthcare, Milwaukee, WI, USA); spiral pitch factor 0.98; collimation 64 × 0.625 mm; and time of rotation 0.6 s. Standard soft tissue reconstruction, by using iterative reconstruction at 40% (ASiR-V, GE Healthcare, Milwaukee, WI, USA), was used for all CT images at slice thickness of 1.25 mm.

### 2.3. CT Scan Morphological Analysis

CT morphological analysis was performed by two readers in consensus (XX and YY, with 5 and 10 years of experience in gastrointestinal oncologic imaging, respectively); the readers were aware of the primary tumor site but not of the pN status. In case of discordances between the two readers, a third super partes reader was considered (ZZ, with more than 20 years of experience in oncologic imaging). They assessed all visible regional LNs according to the primary tumor site: ileocolic and right colic (caecum); ileocolic, right colic, and middle colic (ascending colon); right colic and middle colic (hepatic flexure); right colic, middle colic, left colic, and inferior mesenteric (transverse colon); middle colic, left colic, and inferior mesenteric (splenic flexure); left colic and inferior mesenteric (descending colon); and sigmoid, left colic, superior rectal, inferior mesenteric and rectosigmoid (sigmoid colon) [19].

A NODE-SCORE of malignancy suspicion based on seven different morphological parameters according to the literature was built [10,20,21]. LNs with long-axis diameter ≤2 mm were not considered for the present and following analysis due to small size, which can hinder accurate evaluation. One point for each parameter was assigned in case of suspicious morphologic aspect, while zero points were assigned in case of benignity characteristics as follows: (a) one point for short-axis diameter measured on axial plane ≥4 mm and zero points for short-axis diameter ≤4 mm; (b) one point for diameter ratio >0.8 (short-to-long axis diameter ratio) and zero points for a ratio <0.8; (c) one point for round LN shape and zero points for oval LN shape; (d) one point for internal heterogeneity/central necrosis (mixed attenuation within the LN) and zero points for internal homogeneity attenuation; (e) one point for loss of fatty hilum and zero points if fatty hilum was preserved; (f) one point for irregular outer border and zero points for regular LN border; and (g) one point for perinodal infiltration (irregular and/or indistinct demarcation of the LN) and zero points for absence of perinodal infiltration [22]. After the analysis, a total LN score was obtained by summing up all the morphological parameters (Table 1), and two groups were obtained: LNs with low likelihood (0–2 points) and LNs with high likelihood (3–7 points) of malignancy.

### 2.4. CT Scan Segmentation Analysis

**Figure 1:**
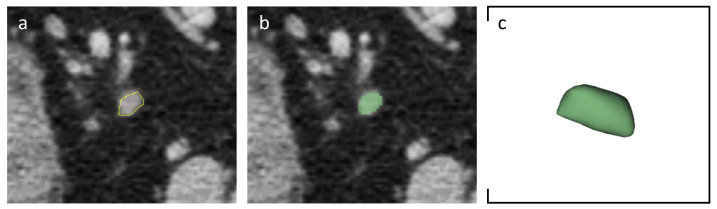
Axial portal-venous phase abdomen CT images from an 84-year-old man with a histologically proven diagnosis of colon cancer. (**a**) Abdomen CT image shows manually-drawn region of interest of a regional lymph node. (**b**) The same image showing the lymph node after radiomic volumetric segmentation (in green). (**c**) The same lymph node rendered in 3D.

The same LNs analyzed with the morphological score were then segmented by using open-source 3D Slicer software (version 4.11.20210226, https://slicer-packages.kitware.com/#item/60add6fdae4540bf6a89bf73 (accessed on 31 January 2024) on portal-venous phase. Slice-by-slice, a volumetric region of interest was manually drawn with the goal of covering total LN’s volume and avoiding perinodal vessels and other organs (Figure 1).

Then, a sub-analysis was performed to answer the second aim of the study. In fact, all primary lesions clearly visible on baseline CT were also segmented in a volumetric manner. For validation cohort, the same segmentation was performed for lymph nodes and primary lesions.

### 2.5. Radiomic Features Extraction

A 3D Slicer radiomics extension (Pyradiomics library [23]) was used to extract 107 radiomic features from both LNs and PLs, including shape, first, and second order features: 19 first order statistics features, 13 2D and 3D shape features, 16 grey level size zone matrix (GLSZM) features, 5 neighboring gray tone difference matrix (NGTDM) features, 14 gray level dependence matrix (GLDM) features, 24 gray level co-occurrence matrix (GLCM) features, and 16 gray level run length matrix (GLRLM) features.

### 2.6. Statistical Analysis

According to NODE-SCORE, LNs were divided into two groups of low and high likelihood of malignancy, respectively. Then, to assess if radiomics was in accordance with pathology rather than NODE-SCORE, we compared radiomic features of LNs with the same grouping in the morphological analysis.

After that, the radiomic features with a *p* value > 0.9 were chosen as features in accordance with the pathology rather than NODE-SCORE; these selected features were compared with the same features extracted from the primary tumor to assess if there were difference between the ultrastructural N0 LNs and primary lesions (PL) in terms of radiomic values. A LN/PL ratio, with no other algorithm superimposed, has been proposed as possible tool to assess LN’s benignity on CT images with a cut-off <0.8 to assess LN’s benignity. As preliminary proof of concept is beyond the primary aim of this study, however, the results obtained with the LN/PL ratio were then tested on the validation cohorts (pN0 and pN2); then, receiver operating characteristic (ROC) curves and multiple tests were performed for both radiomics LN/PL ratio and NODE-SCORE with DeLong method [24].

All data are expressed as mean ± standard deviation (SD). Normal data distribution was assessed by Kolmogorov–Smirnov test. Student *t*-test and Mann–Whitney U test, based on Gaussian normality or non-normality, respectively, were used to compare continuous variables. Categorial variables were reported with numbers and percentages, then compared with χ2 test with or without Yates correction. Correction for multiple comparisons was performed with Holm–Bonferroni method [25]. McNemar test was performed to test the difference between paired proportions.

Statistical significance was assessed with a *p* < 0.05. Statistical analysis was performed with MedCalc (MedCalc Software, version15, Ostend, Belgium).

## 3. Results

### 3.1. Study Population

**Figure 2:**
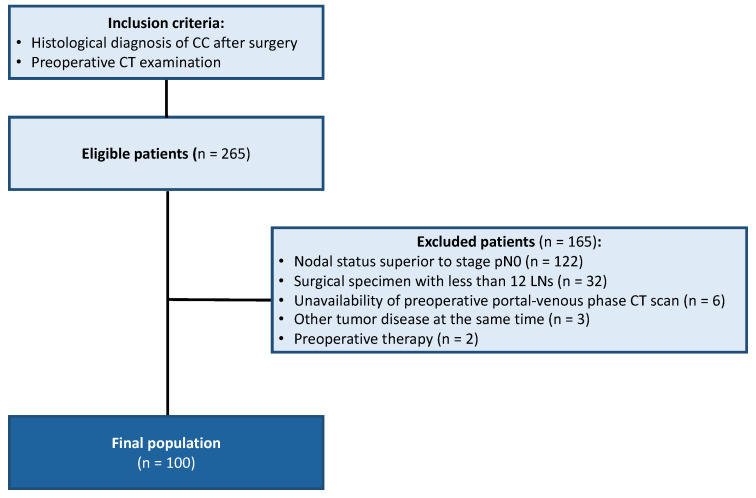
Patients’ enrollment flowchart (test cohort).

Thus, in accordance with the inclusion/exclusion criteria, the final population enrolled consisted of 100 patients with stage I to stage IIC, in particular, 56 males and 44 females (56% and 44%, respectively) with an age range of 41–94 years (median age of 57 years). The enrollment flowchart for the test set of the study is shown in Figure 2.

### 3.2. NODE-SCORE

A total of 337 regional LNs were included in the test cohort. According to NODE-SCORE, LNs were divided into two groups: 167/337 (49.6%) LNs with a low likelihood of malignancy (NODE-SCORE 0–2) and 170/337 (50.4%) LNs with a high likelihood of malignancy (NODE-SCORE 3–7). All morphologic features resulted significantly different between the two groups (all *p* < 0.05, Table 2).

For the test cohorts, a total of 20 LNs were included and 18/20 (90%) reached a HLM NODE-SCORE (NODE-SCORE 3–7) while 2/20 (10%) reached a LLM NODE-SCORE (NODE-SCORE 0–2).

### 3.3. D Segmentation and LN’s Radiomic Features

From the volumetric nodal and PL segmentation, the software itself has routinely extracted 107 radiomic features from portal-venous phase CT scans. After correction for multiple testing, the comparison between LNs with a low likelihood of malignancy and LNs with a high likelihood of malignancy showed 92 radiomic features that were not significantly different. On the other hand, 15 radiomic features were significantly different (Table 3).

In particular, significant differences between the two groups of LNs were found in 10 shape features (elongation least axis length, maximum 2D diameter row, maximum 2D diameter slice, mesh volume, minor axis length, surface area, surface volume ratio, maximum 2D diameter column, and flatness. All *p* < 0.015) and 5 first order features (voxel volume, 90 percentile, total energy, median, and mean. All *p* < 0.018).

No significant difference was found among gray level size zone matrix (GLSZM and *p* > 0.938), gray level run length matrix (GLRLM and *p* > 0.05), gray level dependence matrix (GLDM and *p* > 0.05), gray level co-occurrence matrix (GLCM and *p* > 0.05), and neighboring gray tone difference matrix (NGTDM and *p* > 0.05) features.

In particular, the highest *p*-values (all *p* > 0.05) were found for Idn (in the GLCM), dependence entropy (in the GLDM), and busyness (in the NGTDM).

### 3.4. Lymph Node/Primary Lesion (LN/PL) Ratio

A total of 81 PLs were also identified on the CECT. These were volumetrically segmented and the radiomic features of Idn, busyness, and dependence entropy were extracted. Then, the selected features were compared with the corresponding LNs.

**Figure 3:**
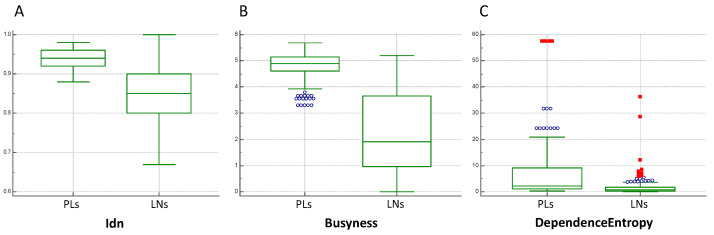
Box plots of estimated correlation values between lymph nodes (LNs) and primary lesions (PLs) of (**A**) Idn, (**B**) busyness, and (**C**) dependence entropy.

The comparison of these last features extracted from a total of 286 LNs and the respective 81 PLs showed statistically significant differences (all *p* < 0.0001, Figure 3).

Finally, as an exploratory intent, a ratio between these three radiomic features of LNs and PLs was obtained and a cut-off <0.8 was considered as an indicator of the LN’s benignity.

**Figure 4:**
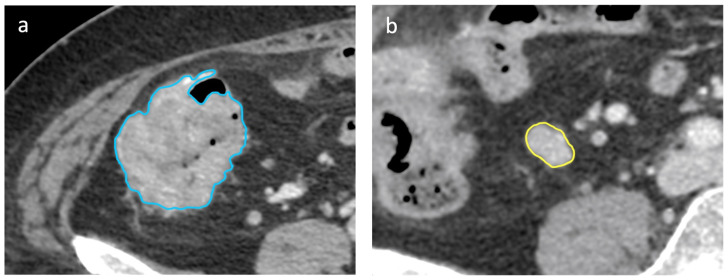
Axial portal-venous phase abdomen CT images from an 89-year-old woman with a histologically proven diagnosis of colon cancer. Abdomen CT image shows manually-drawn region of interest of the (**a**) primary lesion (in blue) and of the (**b**) regional lymph node (in yellow) for radiomic volumetric segmentation.

By so doing, the LNs/PLs ratio correctly identified 19/286 LNs for Idn, 239/286 for dependence entropy, and 213/286 for busyness. This compared with a LN NODE-SCORE that correctly identify 138/286 LNs as LLM in accordance with the pathology (all *p* < 0.001), Figure 4.

### 3.5. Validation Cohorts

**Figure 5:**
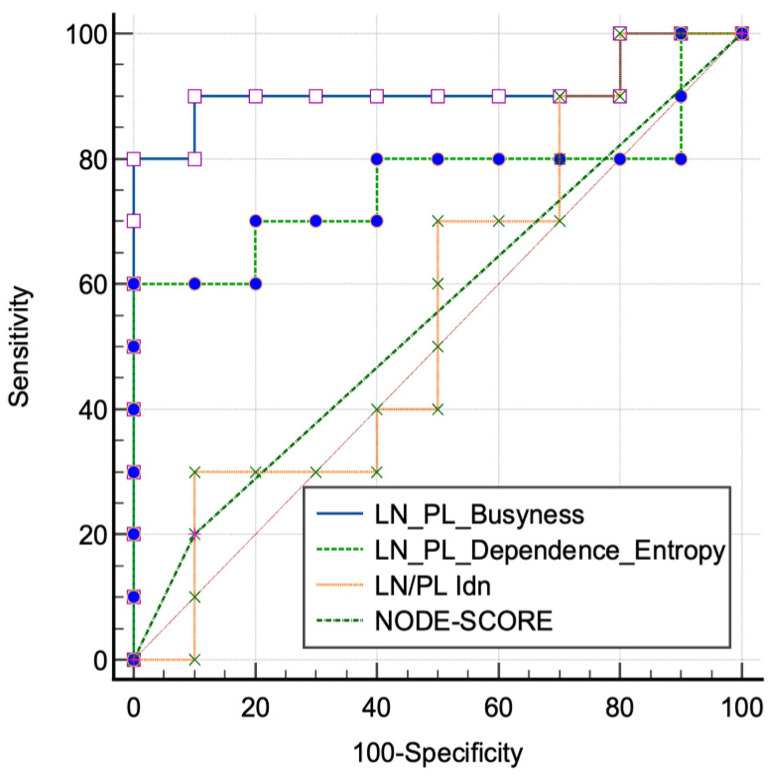
Receiver characteristic operating (ROC) curves tested on the validation cohorts of lymph node (LN) and primary lesion (PL) ratio extracted from the significant features of the training cohorts and from the morphological NODE-SCORE.

**Figure 6:**
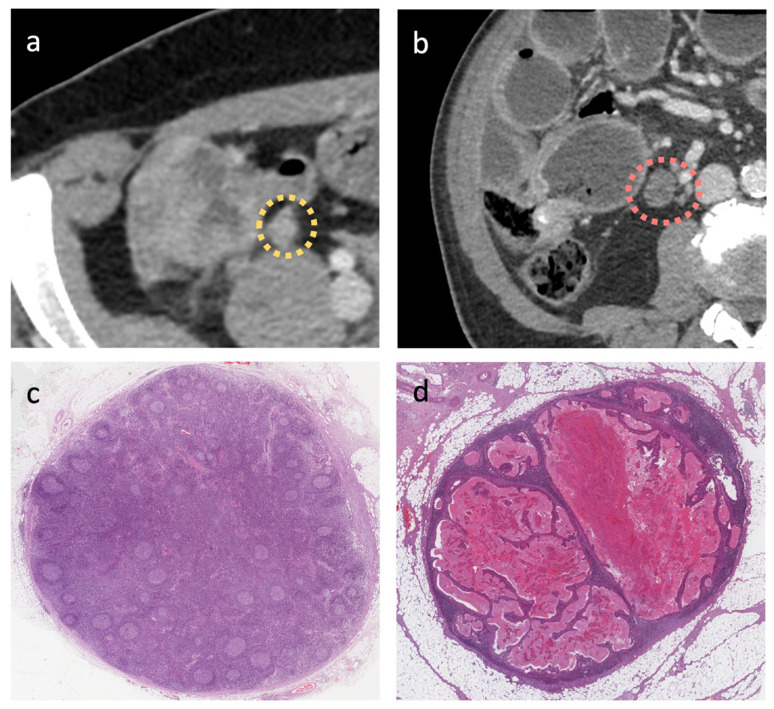
Examples of enlarged lymph node (LN) on CT images (axial porta-venous phase) in (**a**,**b**); both are highly suspected to be malignant according to the size, round shape, and irregular margins. In (**c**,**d**), the pathology images show the reactive benign and malignant LNs, respectively, (H and E stains and low magnification).

External validation cohorts showed significant results for ROC curves of radiomics for LN/PL obtained from the busyness feature, with an AUC of 0.91 (0.69–0.99, 95% C.I., *p* < 0.001, sensitivity 90%, and specificity 90%); significant results have also been obtained for LN/PL from the dependence entropy feature, with a AUC of 0.76 (0.52–0.92, 95% C.I., *p* = 0.03, sensitivity 60%, and specificity 100%). Non-significant results were obtained for LN/PL derived from Idn, with an AUC of 0.56 (0.32–0.77, 95% C.I., *p* = 0.66, sensitivity 100%, and specificity 20%). Non-significant results were also obtained for the NODE-SCORE, with an AUC of 0.55 (0.35–0.76, 95% C.I., *p* = 0.54, sensitivity 20%, and specificity 90%), Figure 5. In addition, representative images of the radiology and pathology of the benign and malignant LNs are appreciable on Figure 6.

## 4. Discussion

The intent of our study was to analyze whether radiomics could better characterize benign lymph nodes in patients with colon cancer than CT morphological features could. The results obtained showed that certain radiomic parameters characterize the internal structure of benign lymph nodes even when morphological features would suggest malignancy. In fact, it is interesting to underline how all the second order features’ results are non-significant between the two groups, in accordance with the pathology. An example is provided by the feature of busyness that measures the change from a pixel to its neighbor: a high value for this parameter is related to an abrupt of intensities between pixels and contiguous neighborhood [23]. In our study, this aspect is clearly appreciable, firstly with the non-significant difference between all LNs in accordance with the pathology and then with the lower value of busyness for LNs (1.46 ± 3.03) compared with PLs (6.25 ± 8.79), which are characterized by high busyness, as expected in tumor lesions. On the contrary, significant differences were found for some shape features and first order features that enhance the volumetric and morphologic parameters of the LNs, similarly to the node-score.

A few studies have analyzed directly the radiomics of LNs due to the difficulty of direct labeling among LNs on imaging and on pathology. Considering this limitation, Li M and colleagues proposed a model that included both radiomics from primary colorectal cancer lesions and peri lymph node tissue. They analyzed a total of 766 patients divided into training and test cohorts and their results show how the decision curve analysis, including a total of six radiomic features from both primary lesions and peripheral lymph nodes, would help clinicians with the preoperative prediction of risk [15]. Despite there being differences to our study, this paper shows how the radiomic characterization of primary tumors and lymph node environment is helpful to assess, before the pathology report, the individual risk factors and to plan the most personalized surgical treatment with a curative intent by avoiding unnecessary overtreatment.

In this scenario, the intrinsic difficulty with matching the exact LN on the pathology representation is an important bias that limits the study of the lymph nodes’ ultrastructural characteristics. In fact, to characterize preoperatively the N status with more precision would help not only CC patients but also several other oncologic districts. For instance, an interesting study conducted on bladder cancer by Starmans M P A et al. [26] showed how radiomics showed no improvement in the discrimination between N0 and N+. Among the possible explanations found by the authors, there is a bias in the N+ feature characteristics that also includes benign lymph nodes due to the lack of direct labelling on the pathology, and then there is the possibility of missing pathologic lymph nodes measuring less than 3 mm due to the intrinsic limits of the CT slice thickness. In accordance with our study, the authors underline how the model based on the largest LNs did not improve the performance of the model itself, as demonstrated by our morphological analysis that included approximately 50% of LNs as having a likelihood of being malignant.

After the demonstration of significant differences between the selected radiomic features of LNs compared with PLs, we proposed a possible easy-to-use application expressed by a ratio between the value of the LN and the PL. This evaluation discriminates negative LNs better than morphologic NODE-SCORE for dependence entropy and busyness. The idea of a ratio between the two entities, which is able to discriminate between healthy homogeneous tissue and malignant heterogeneous tissue, relies on similar concept obtained in nuclear medicine. Example are provided by Cho J et al. [27], with higher performances of the SUV ratio between LNs and PLs, and by Chen P J and colleagues [28] that demonstrated the better performances of a SUV ratio between LNs and PLs in esophageal cancer in terms of survival.

After the radiomics features selection, the validation cohorts were used to test the radiomics LN/PL ratio and the NODE-SCORE in order to discriminate between pN0 and pN2 status. The results obtained showed an interesting performance of the LN/PL ratio derived from busyness, with an AUC of 90% with sensitivity of 80% and specificity of 100%; on the contrary, the NODE-SCORE did not reach significant results, confirming the limitation of the morphological assessment for LN’s characterization. In this scenario, an interesting metanalysis showed how the CT accuracy in detecting malignant LNs in CC had a sensitivity, specificity, and diagnostic odds ratio (DOR) of 70% (95% CI: 63–73%), 78% (95% CI: 73–82%), and 8.1 (95% CI: 4.7–14.1), respectively [29]. Our results showed how radiomics might improve the accuracy of the LN’s characterization over the morphological characteristics.

Despite the interesting results of our preliminary study, there are several limitations. Firstly, the retrospective nature of the study; then, the difficulty with performing an accuracy analysis or a performance analysis due to the lack of positive LNs to analyze in the test cohort. In addition, the characteristics of the validation cohorts were retrospective, small, and with a lack of direct comparison with the pathology even if the choice of pN2 status assured a discrete level of certainty. Finally, an integration of our data with artificial intelligence approaches is lacking and might improve the integration of data derived from radiomics, clinical data, and CT semantic features.

These aspects might be overcome in a future study where the possibility of identifying the exact lymph nodes assessed in CT scans with those analyzed by pathologists can be explored. By so doing, it will be possible to test the data emerging from this preliminary analysis and to assess the performances of radiomics and the LN/PL ratio with a direct comparison of both benign and malignant LNs in a pathology specimen. In addition, the present study also lacks a combined model including clinical and radiomics data to assess if the performances, by merging different fields, might improve the risk stratification in CC patients.

## 5. Conclusions

In conclusion, our study demonstrates the potentiality of radiomics for the characterization of N0 LNs in CC over morphologic assessment; the possibility of radiomic features able to characterize benign LNs might help in the future in the risk stratification of CC patients before treatment planning, in order to obtain a treatment plan personalized for the patient. Further studies are needed.

## Figures and Tables

**Table 1:** Lymph node-score based on CT morphological imaging.

Morphological Imaging		Point Score
Size		
Short axis	Enlarged (≥4 mm)	1
	Normal (<4 mm)	0
Configuration		
Diameter ratio	≥0.8 mm	1
	<0.8 mm	0
Shape	Round	1
	Oval	0
Texture	Heterogeneous / Focal necrosis	1
	Homogeneous	0
Fatty hilum	Loss	1
	Preserved	0
Border	Irregular	1
	Smooth	0
Perinodal infiltration	Yes	1
	No	0

**Table 2:** LN’s morphological assessment findings. * The chi-square statistic with Yates correction.

Morphologic Features	Low Likelihood of Malignancy (n = 167)	High Likelihood of Malignancy (n = 170)	*p*-Value
Short axis (≥4 mm)	51 (30.5%)	162 (95.3%)	<0.00001
Diameter ratio (≥0.8 mm)	48 (28.7%)	119 (70%)	<0.00001
Shape (round)	29 (17.4%)	95 (55.9%)	<0.00001
Texture (Heterogeneous)	3 (1.8%)	69 (40.6%)	<0.00001
Fatty hilum (loss)	76 (45.5%)	167 (98.2%)	<0.00001
Border (irregular)	6 (3.6%)	20 (11.8%)	<0.00494 <0.00914 *
Perinodal infiltration (yes)	17 (10.2%)	77 (45.3%)	<0.00001

**Table 3:** Radiomic features. * Holm–Bonferroni correction.

Radiomic Features (n = 107)	Low Likelihood of Malignancy (Mean ± SD)	High Likelihood of Malignancy (Mean ± SD)	*p*-Value	Adjusted *α*-Value	*p*-Value *
SHAPE (n = 13)					
Elongation	0.67 ± 0.20	0.74 ± 0.19	0.0001	0.00047	0.005
Least Axis Length	2.11 ± 1.82	3.17 ± 2.25	0.0001	0.00047	0.0049
Maximum 2D Diameter Row	6.19 ± 2.05	7.18 ± 2.12	0.0001	0.00048	0.0048
Maximum 2D Diameter Slice	6.02 ± 2.05	7.21 ± 2.73	0.0001	0.00048	0.0047
Mesh Volume	61.32 ± 51.38	120.02 ± 129.74	0.0001	0.00049	0.0046
Minor Axis Length	4.39 ± 1.56	5.45 ± 1.70	0.0001	0.00050	0.0045
Surface Area	86.43 ± 47.97	129.43 ± 89.51	0.0001	0.00050	0.0044
Surface Volume Ratio	1.79 ± 0.63	1.42 ± 0.45	0.0001	0.00050	0.0043
Maximum 2D Diameter Column	6.24 ± 2.08	7.31 ± 2.56	0.0003	0.00052	0.0117
Flatness	0.32 ± 0.28	0.42 ± 0.29	0.0004	0.00053	0.0152
Others	-	-	>0.0006	>0.00054	>0.021
FIRST ORDER (n = 19)					
Voxel Volume	93.31 ± 71.50	165.09 ± 154.93	0.0001	0.00051	0.0042
90 Percentile	69.01 ± 119.94	91.63 ± 22.85	0.0001	0.00051	0.0041
Total Energy	758,294.13 ± 3,100,934.06	855,254.5 ± 1,076,742.65	0.0001	0.00052	0.004
Median	37.8 ± 117.56	60.12 ± 27.23	0.0004	0.00053	0.0148
Mean	36.48 ± 116.98	57.95 ± 24.99	0.0005	0.00054	0.018
Others	-	-	>0.0008	>0.00055	>0.0272
GLSZM (n = 16)					
All	-	-	>0.0335	>0.00059	>0.938
GLRLM (n = 16)					
All	-	-	>0.058	>0.00060	>1.5444
GLDM (n = 14)					
All	-	-	>0.0849	>0.00061	>2.1225
GLCM (n = 24)					
All	-	-	>0.1218	>0.00063	>2.6796
NGTDM (n = 5)					
All	-	-	>0.3307	>0.00109	>2.7523

## Data Availability

The data presented in this study are available on request from the corresponding author.
